# Supplementary figures and images for: Topology across scales on heterogeneous cell data
Source: PLoS Comput Biol. 2025 Oct 15;21(10):e1013460. doi: 10.1371/journal.pcbi.1013460 (PMC12527197; doi:10.1371/journal.pcbi.1013460)

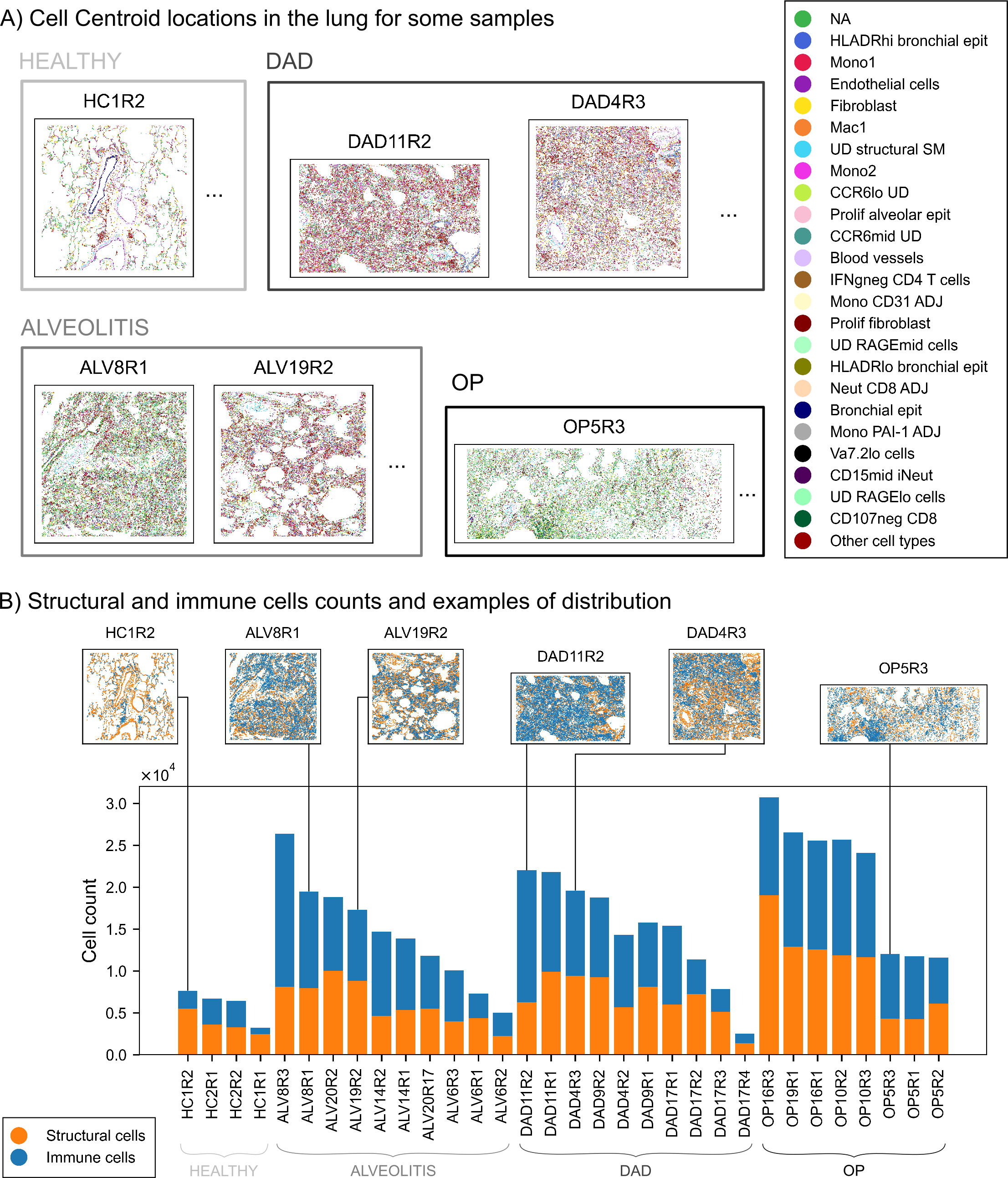

Supplement: S1 Fig — A: Spatial distribution of cell centroids for the 25 most common cell types (out of 50) in the COVID-19 human lung IMC data set, shown for six representative samples out of 32 total. The data set includes 32 lung sections, categorised by disease stage. We display one of the four healthy samples, two of the ten in the alveolitis (ALV) stage, two of the ten in the diffuse alveolar damage (DAD) stage, and one of the eight in the organising pneumonia (OP) stage. B: Cell counts of the two broader types of cells: structural and immune, for the 32 samples in the data set, together with the spatial distribution of the two types of cells in the six representative samples from above. Note that the sizes of the samples vary. (TIF) [file pcbi.1013460.s001.tif]

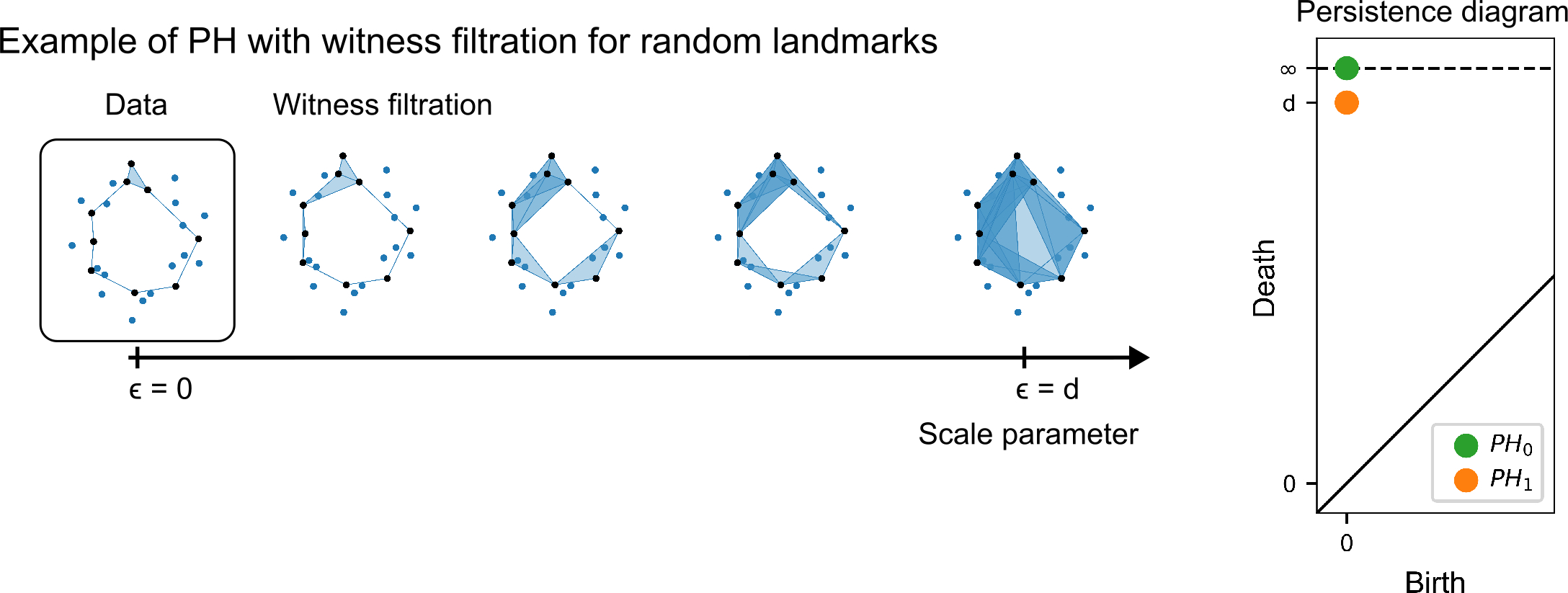

Supplement: S2 Fig — Example of the witness filtration for a point cloud forming a loop. Landmarks points, in black, are chosen randomly. The persistence diagram contains only one point in each degree, corresponding to a single connected component and the loop, both formed at ϵ=0. (TIF) [file pcbi.1013460.s002.tif]

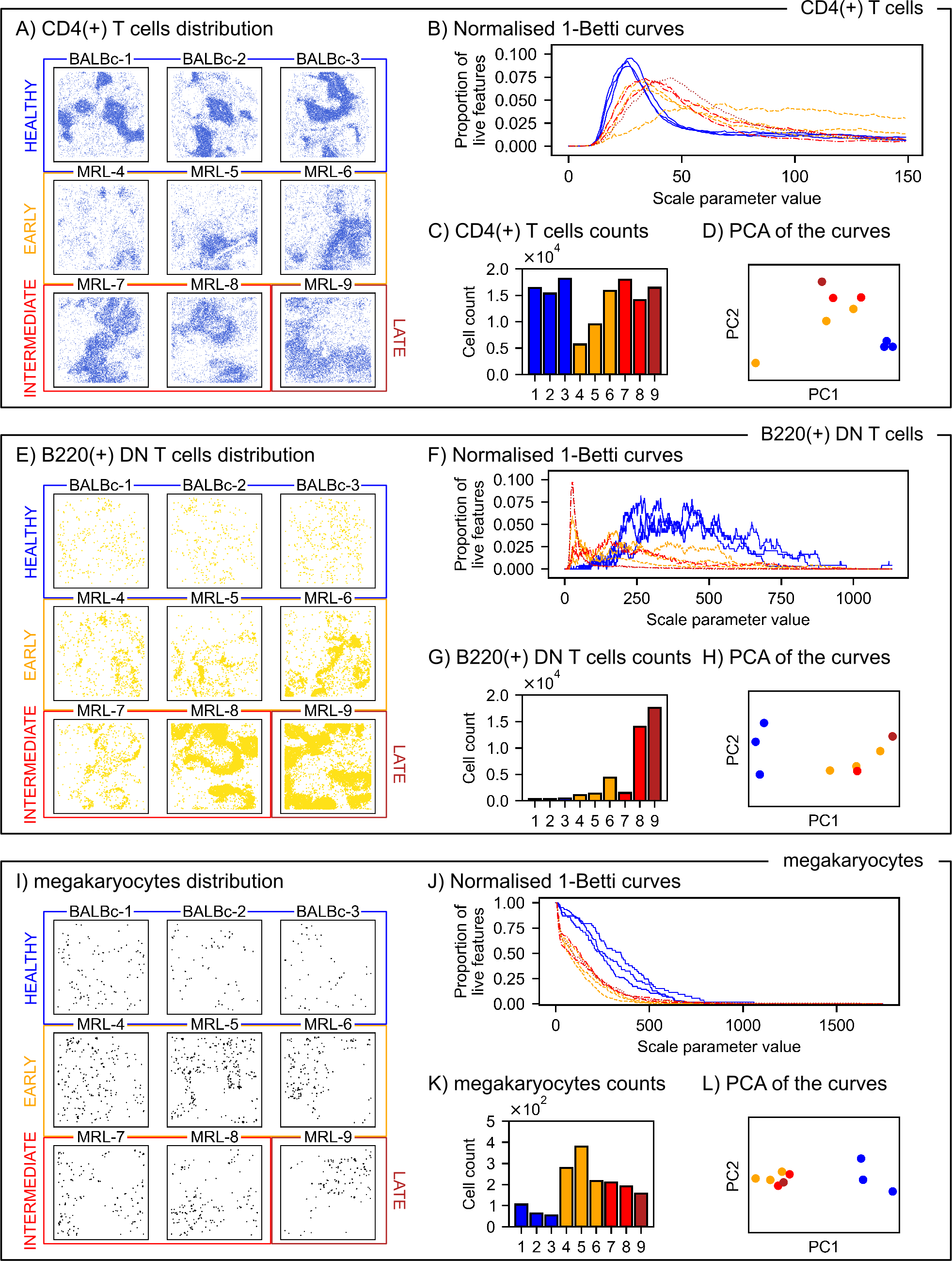

Supplement: S3 Fig — A: CD4(+) T cells locations in the lupus murine spleen CODEX data. B: CD4(+) T cells normalised Betti-1 curves. The differences in the location of the peak indicate an decrease of density within the dense regions of the CD4(+) T cells in disease. One of the early diseased samples shows a behaviour different from the rest, due to the absence of dense regions. C: Cell counts of the CD4(+) T cells for each sample. For this cell type, the total cell counts do not change significantly between health and disease (Welch t-test p-value is 0.16). D: PCA plot of the CD4(+) T cells normalised Betti curves of degree 1 of the alpha filtration. E, F, G, H: same plots for B220(+) DN T cells. In this case the change in the location of peaks happens gradually as the disease progresses, due to the appearance and growth of large dense regions. We observe multiple peaks in the early diseased curves. I, J, K, L: same plots for megakaryocytes, but for the case of Betti-0 curves, which count the proportion of clusters which are alive at a given parameter value. We observe a very sharp decrease in the disease curves at small values, which is due to the presence of clusters in the cell population cells. (TIF) [file pcbi.1013460.s005.tif]

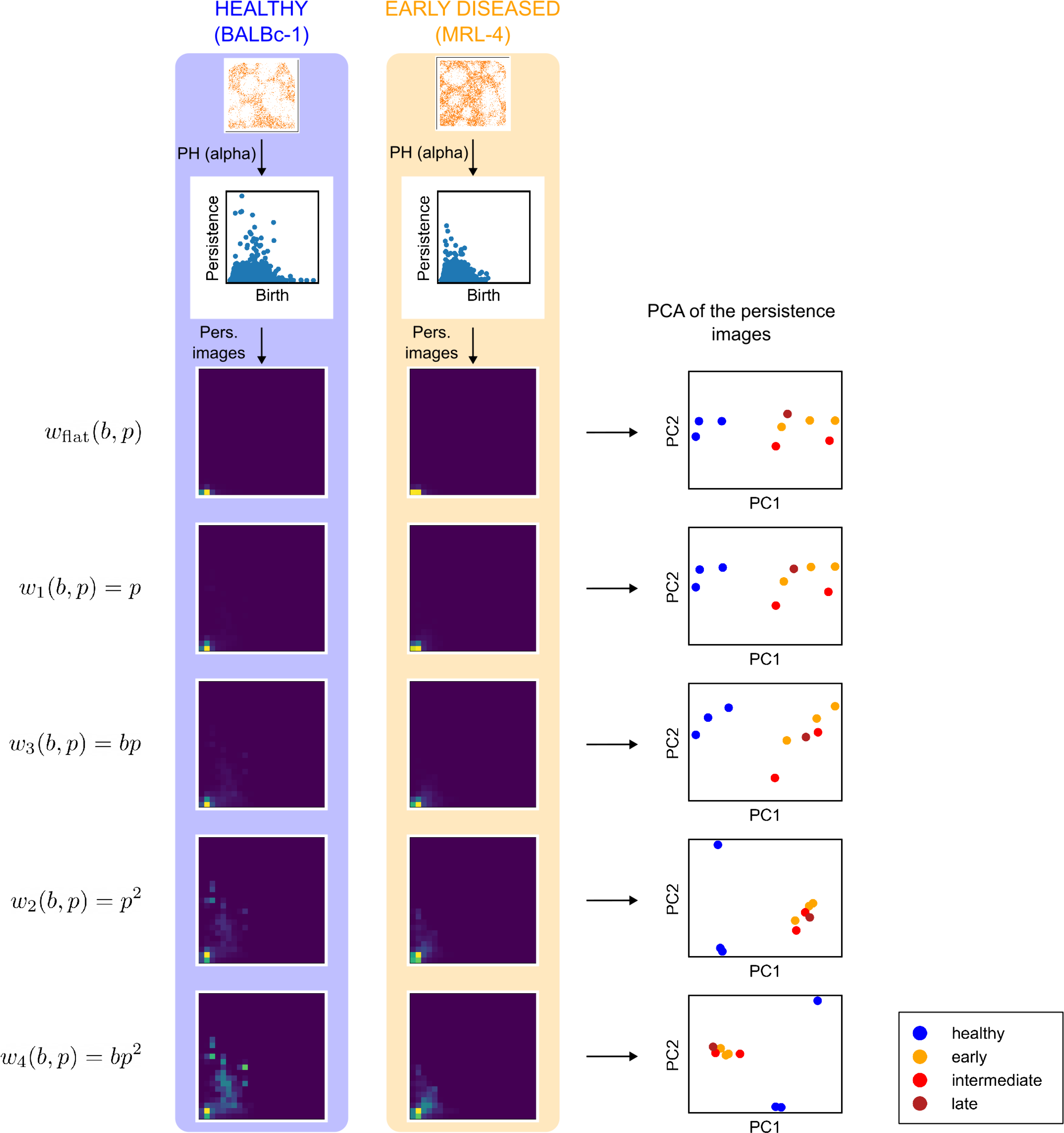

Supplement: S4 Fig — Examples of persistence images with five different weighting functions for the red pulp with alpha filtration (BALBc-1, MRL-4). Here we observe how weights that depend on persistence, p, and/or birth value, b, “see" the large-scale features in the diagram. For the case of red pulp, the presence of a large number of small-scale features causes w1 to yield very similar images to wflat. A more intense weighting on persistence, like w3 or w4, is needed in order to highlight the large-scale. We include the PCAs of the persistence images for each weighting function. (TIF) [file pcbi.1013460.s006.tif]

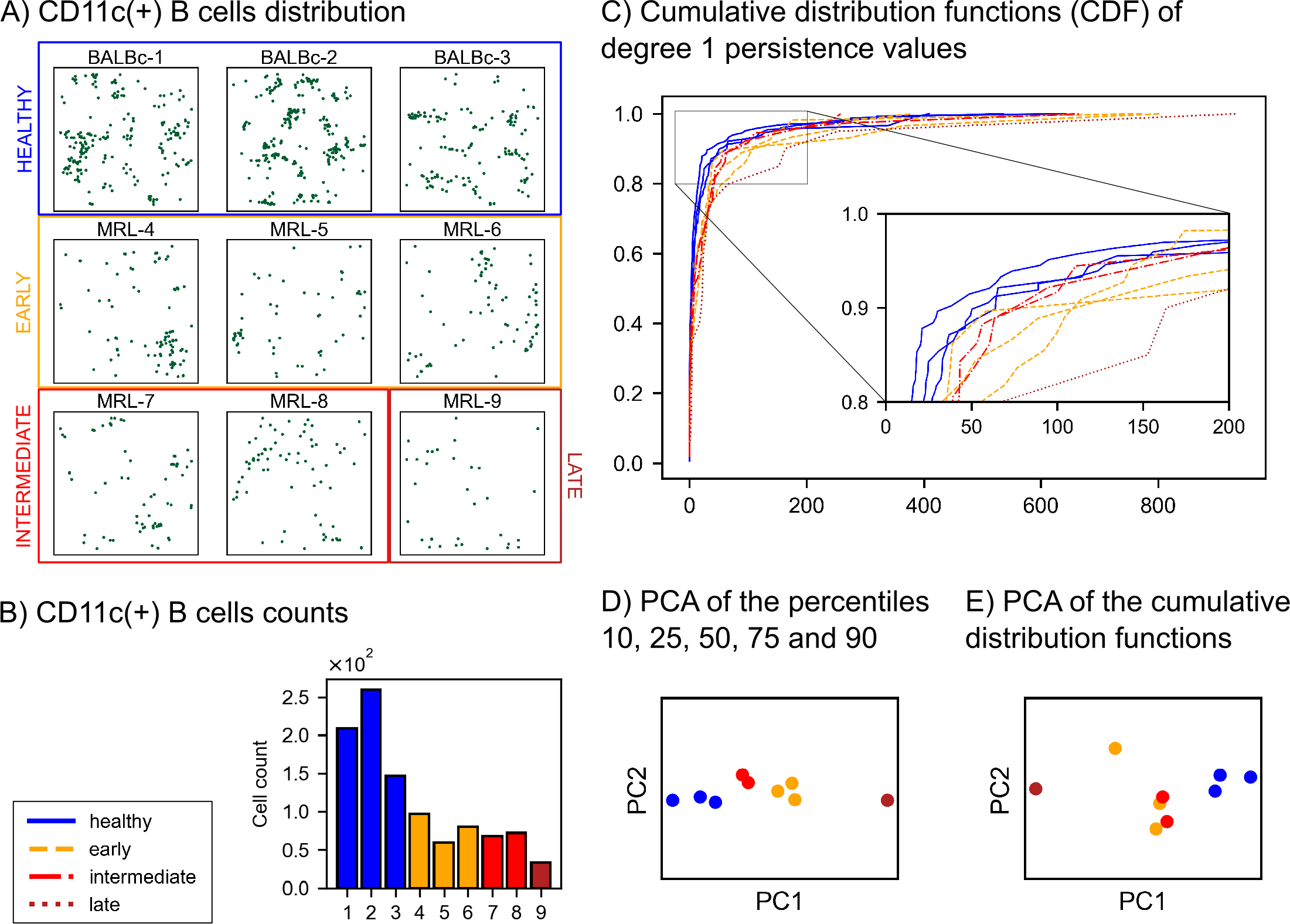

Supplement: S5 Fig — A: CD11c(+) B cells locations in the lupus murine spleen CODEX data. We see this cell type forms denser regions in the healthy samples, and is more sparsely distributed in the diseased ones. This is detected by three topological descriptors of degree 0, which yield correct 2-clusterings (see last rows of S3 Table). However, there is no visually clear distinction between early and intermediate samples. B: Cell counts of the CD11c(+) B cells for each sample. C: To further study the distribution of persistence values, we plot the cumulative distribution function of degree 1 persistence values for CD11c(+) B cells. Visually, these curves do not form 4 distinct groups, corresponding to the 4 stages. We note, however, that on the 90th percentile (when curves hit value 0.9) the three early stage curves are much closer and separated from the two intermediate stage curves. D: PCA plot of the CD11c(+) B cells topological descriptor obtained from the 10th, 25th, 50th, 75th and 90th percentiles of degree 1 persistence values, whose 4-clustering coincides with the stages of the disease. We observe that clusters are separable by their PC1 coordinates, and we check that the PC1 component is dominated by the 90th percentile (with a weight of 0.96). Hence, the coincidence of the early diseased curves on the 90th percentile we observed in B explains the clustering result. E: PCA plot of the CD11c(+) B cells topological descriptor obtained from cumulative distribution functions in B. To show that the cumulative distribution functions do not in fact separate in 4 groups, we use 4-means clustering directly on the curves. We obtain clusters that separate healthy and late stages correctly from the rest, but mix early and intermediate curves. We conclude that the correct 4-clustering with percentiles was an artifact of the choice of high percentiles, which are unstable for small populations. (TIF) [file pcbi.1013460.s007.tif]
