# Supplementary material for: Topology across scales on heterogeneous cell data
Source: PLoS Comput Biol. 2025 Oct 15;21(10):e1013460. doi: 10.1371/journal.pcbi.1013460 (PMC12527197; doi:10.1371/journal.pcbi.1013460)
Supplement: Attachment — Submitted filename: Response to Reviewers.pdf [file pcbi.1013460.s011.pdf]

August 21, 2025

PLOS Computational Biology

Dear Dr. Copos and Dr. Finley,

Thank you for considering our manuscript for publication in PLOS Computational Biology. We thank the reviewers for their valuable feedback and have revised the manuscript in line with their comments. Changes in the manuscript are marked in blue.

The three main improvements to the manuscript are (1) biological interpretation of the results, (2) clarification about data and definitions, and (3) correct errors/typos.

(1) For biological interpretation: We have incorporated additional text in the Discussion (p. 23) to explain how our main quantitative findings align with previous pathology studies on structural changes in the lupus spleen and COVID-19 human lungs, as suggested by reviewer #2. We have focussed on comparing our results to the previous analyses of the same data sets [1, 2]. We have also reanalysed the positive result for the CD11c(+) B cells in the lupus spleen, which was not discussed in the previous version of the manuscript as noted by reviewer #1. We have incorporated additional text in the Results section (p. 17) and a new Figure S5 in the SI and accompanying captions to give a more complete account of the result.

(2) For clarification of data and definitions: We now restate the definition of "correct clustering" in the Results section (p. 16) as suggested by reviewer #1. We have included a summary of the second data set (SI Figure S1) to orient the reader, as suggested by reviewer #2. We also now state the average number of points per point cloud across samples in the results tables S3, S4 and S5 in the SI, as suggested by reviewer #1. We also added a new table (SI Table S1) detailing the proportion of randomly chosen landmark points in witness filtrations for each point cloud, and a reference in the Methods section (p. 3).

(3) Correct errors/typos: We have corrected an error in Figure 8 (p. 22). In six samples, the images showing the cell centroid distribution for Endothelial cells were incorrect; they instead depicted the distribution for all cells in the sample. This affected the samples used in Figure 7: ALV8R1, ALV19R2, DAD4R3, DAD11R2, and OP5R3. We have also corrected an error in Figure S3, where megakaryocytes where incorrectly labeled as B220(+) DN T cells. The caption and figures were correct.

In conclusion, the reviewers' helpful comments have enabled us to greatly improve our manuscript. In what follows, we explain how each of the reviewers' comments have been addressed.

Thank you for consideration and we believe it is now suitable for publication.

Best wishes,

Heather Harrington (on behalf of all authors)

Reviewer #1

Comment 1: Add number of points in each point cloud
I would like the authors to include one more data field in Tables 2,3,4: could they please add a column with the number of points in each point cloud, and the number of witness points used when building the witness filtrations.

Response: In tables 3, 4 and 5 (formerly 2, 3 and 4, p. 30-32), we now state the average number of cells of each cell type across samples, and the average number of landmark points (if witness complexes are computed for that cell type). This information is included as part of the "Point cloud" (cell type) column rather than in a separate column, to improve readability. We have also included a new table in the SI (Table S1), detailing the proportion of randomly chosen landmark points in witness filtrations for each point cloud; we include a reference to the new Table 1 in the Methods section (p. 3)

Comment 2: Clarify meaning of "correct clustering"
On page 16, I would like the authors to clarify what they mean by "correct clustering"? Does this mean all labelled data are correctly allocated to a respective cluster with respect to a given TDA vectorisation? (this seems too strong). Or is it that the silhouette score is positive? (this seems rather generous).

Response:

We have now restated the definition of "correct clustering" in the Results section (p. 16) for clarity and improved interpretation. (The definition of "correct clustering" appears in the Methods section on p. 13.)

By "correct clustering", we mean that the clustering obtained using a given TDA vectorisation exactly matches the biological ground truth labels. In the case of 2-clustering, this means a perfect separation between healthy and diseased samples; for 4-clustering, it means that the clusters exactly match the four disease stages (e.g. healthy, early, intermediate, and late in the lupus data set). All vectorisations listed in Tables 2 and 3 meet this strict criterion. The silhouette score is reported separately and serves to assess the internal quality of the clustering.

We agree that correct clustering is a strong condition. However, since the lupus data set contains only 9 samples, even a single misclassified point significantly reduces clustering accuracy. Therefore, we chose to focus on exact matches in that setting. For the COVID-19 data set, which is larger (32 samples), we also looked at clusterings which partially agreed with the biological classification, as measured by the Rand index (Table 4).

Comment 3: No discussion of the second cell type that gave a correct 4-clustering in the lupus data set
Also in that section, there is no discussion of the second cell type that gave a correct 4-clustering of the lupus samples (CD11c(+) B cells) using a statistical percentile of persistence vectorisation. The score for this 4-clustering is 0.4996, which is stronger than that for the plain B cells with Betti-1 curve vectorisation with 0.3319. Could the authors comment on this?

Response:

We agree with the reviewer and we have now looked more carefully at this result.

The second cell type, CD11c(+) B cells, is a sparse cell type which accounts for between 33 and 260 cells per sample, making up for less than 0.2% of the total number of cells. Given the cell sparsity, we did not further explore the statistical significance of the spatial arrangement to disease progression. The correct 4-clustering is obtained from the percentiles of persistence values of degree 1. By looking at the cumulative distribution function of persistence values, we found that the correct result is a consequence of the choice of the 90th percentile. Since it is in the tail of this distribution, changing to the 85th or 95th changes the clustering results. For other cell types, this percentile would not be in the tail; therefore the sparsity makes this clustering relatively unstable and statistically insignificant.

We have incorporated additional text in the Results section (p. 17) and a new Figure S5 in the SI and accompanying captions to describe this in detail.

Comment 4: Incomplete sentence in page 16
A sentence on page 16 seems incomplete/ not properly integrated with the text: "Two cell types that give a significant spatial pattern between disease stages."

Response: We thank the reviewer for spotting this typo. The text now reads: "Two cell types show significantly different spatial patterning between disease stages: B cells and CD11c(+) B cells (a sparse cell type)."

Comment 5: "Persistent homology" instead of "Persistence homology"
The abstract has "persistence homology" but the standard term is "persistent homology".

Response: We thank the reviewer for spotting this inconsistency which we have now corrected in the abstract and throughout the manuscript.

Comment 6: Convention for numbers in text
Check the journal style preference regarding the convention that numbers smaller than ten are written as words and those larger as digits? eg I would rewrite "We next analyse all 25 cell types and 2 broader groups of cell types" as "We next analyse all 25 cell types and two broader groups of cell types", but later in that paragraph keep "2-clustering" as is.

Response: We have not found a specific convention for numbers in the journal's submission guidelines, but we have standarised the text as you propose: words for numbers up to ten, and digits for larger numbers, with the exception of the more technical terms, such as "2-clustering" or "degree 1 homology".

Reviewer #2

Comment 1: Summary of second data set
To demonstrate this idea [PWDS visualisation] they use two previously published spatial datasets, which come with annotations. One of the data sets is summarised in Figure 2 which helps orient the reader but I could not see a similar summary of the other data. Perhaps that is something to include for completeness.

Response: We added Figure S1 in the SI with a summary of the second data set for completeness. Since this data set is larger than the first data set (32 samples) we include only a subset of representative examples.

Comment 2: Biological insight provided by the results
The main conclusions are quite technical however, and my main query concerns the additional insight that their analysis provides.

Do the topological features they identify only serve to highlight characteristics of the diseases under study that were already known (i.e. are the results a mathematical way of saying what a pathologist would already know)?

- If yes then do they shed any extra light on those characteristics (could they provide quantitative metrics that might help practitioners interpret pathology images, for example)?
- If no (i.e. their analysis uncovers biological characteristics that were unknown), then are those characteristics amenable to further exploration?

I think that while technically excellent the paper would be improved with some more discussion of the biological meaning of their analysis and how it relates to what is already known about the diseases under study.

Response:

Detailed analyses of the spatial distribution of different cell populations in lupus spleen and COVID-19 lungs have been undertaken by other authors (see [3, 4] and [5, 6] for details). Our paper provides quantitative, multiscale descriptions of structures that can be identified in 2D images and are consistent with the qualitative results and measurements based on local environments from these previous studies.

We now have included additional text in the Discussion (p. 23) which explains how our main quantitative findings align with previous studies, focussing on existing analyses of our data sets [1, 2]. For completeness, we details these points below.

The topological visualisation and computations align with known spatial rearrangements of red and white pulp cell types (1-3), B cell follicles (4), and other individual cell types (5-7) in the lupus murine spleen, while also offering new perspectives on these changes. The method detects clusters of megakaryocytes (8), a feature not previously reported in [1].

1. Dissipation of marginal zone. The PWDS visualisation highlights the increase of red pulp cells in the white pulp in disease (Figure 5), quantified by persistence diagrams and persistence images. These results align with the dissipation of the marginal zone that separates red and white pulp quantified in [1, 7].

2. Invasion of red pulp with erythroblasts. Betti curves quantify the increase in red pulp cell density with disease (Figure 6B), which aligns with known invasion of red pulp with erythroblasts [1].

3. Disintegration of the PALS (part of the white pulp). Betti curves quantify the decrease in density of white pulp cells within the white pulp (Figure 6F), which matches reports of the PALS disintegration during lupus progression [1].

4. Evolution of B follicles. Betti curves capture the gradual decrease first in density and later in size of B cell populations in lupus (Figure 6J), resulting in a clustering that reflects disease stage. This aligns with and expands the findings of [1], which reported a reduction in the number of B cell rings, a structure typically found in healthy follicles.

5. CD4(+) T cells decrease. Betti curves quantify the decrease in density of CD4(+) T cells (SI Figure S3B). This finding aligns with the dissipation of the PALS found in [1].

6. B220(+) DN T cells increase. We confirm the increase in B220(+) DN T cells observed in [1]. We offer a finer description of their spatial distribution with Betti curves (SI Figure S3F).

7. Dissipation of marginal zone macrophages. Persistence images obtained from the distribution of marginal zone macrophages cluster according to healthy and diseased, which matches the dissipation of the marginal zone observed in [1, 7].

8. Formation of megakaryocyte clusters. We detect clustering of megakaryocytes in disease (SI Figure S3J), a feature not reported in [1]. An increase in the number of megakaryocytes was observed in [7].

For the COVID-19 human lungs data set:

- COVID-19 lung infiltration. Topological descriptors captures changes in the gross infiltration patterns in healthy and diseased lungs (Figure 7), which are consistent with established lung pathology [5, 6].

- Endothelial cell topology. We find that Endothelial cell topology aligns best with clinical staging of COVID-19 lungs (Figure 8), suggesting a new area of focus not explored in the co-localisation studies in [2].

References

[1] Yury Goltsev et al. "Deep Profiling of Mouse Splenic Architecture with CODEX Multiplexed Imaging". In: *Cell* 174.4 (Aug. 2018), 968–981.e15. DOI: 10.1016/j.cell.2018.07.010. URL: https://doi.org/10.1016/j.cell.2018.07.010.

[2] Praveen Weeratunga et al. "Single cell spatial analysis reveals inflammatory foci of immature neutrophil and CD8 T cells in COVID-19 lungs". In: *Nature Communications* 14.1 (Nov. 2023). ISSN: 2041-1723. DOI: 10.1038/s41467-023-42421-0. URL: http://dx.doi.org/10.1038/s41467-023-42421-0.

[3] Birgit Lieberum and Klaus-Ulrich Hartmann. "Successive changes of the cellular composition in lymphoid organs of MRL-Mplpr-lpr mice during the development of lymphoproliferative disease as investigated in cryosections". In: *Clinical Immunology and Immunopathology* 46.3 (1988), pp. 421–431.

[4] Bruce A. Jacobson et al. "Anatomy of autoantibody production: Dominant localization of antibody-producing cells to T cell zones in fas-deficient mice". In: *Immunity* 3.4 (1995), pp. 509–519.

[5] Luca Carsana et al. "Pulmonary post-mortem findings in a series of COVID-19 cases from northern Italy: a two-centre descriptive study". In: *The Lancet Infectious Diseases* 20.10 (Oct. 2020), pp. 1135–1140. ISSN: 1473-3099. DOI: 10.1016/s1473-3099(20)30434-5. URL: http://dx.doi.org/10.1016/s1473-3099(20)30434-5.

[6] Thomas Menter et al. "Postmortem examination of COVID-19 patients reveals diffuse alveolar damage with severe capillary congestion and variegated findings in lungs and other organs suggesting vascular dysfunction". In: *Histopathology* 77.2 (July 2020), pp. 198–209. ISSN: 1365-2559. DOI: 10.1111/his.14134. URL: http://dx.doi.org/10.1111/his.14134.

[7] Qian Zhang et al. "Predominant Role of Immunoglobulin G in the Pathogenesis of Splenomegaly in Murine Lupus". In: *Frontiers in Immunology* Volume 10 - 2019 (2020). ISSN: 1664-3224. DOI: 10.3389/fimmu.2019.03020. URL: https://www.frontiersin.org/journals/immunology/articles/10.3389/fimmu.2019.03020.
